# Influences of* Corydalis decumbens* on the Activities of CYP450 Enzymes in Rats with a Cocktail Approach

**DOI:** 10.1155/2019/9614781

**Published:** 2019-01-21

**Authors:** Chen Cheng, Jianchang Qian, Zhe Wang, Wanshu Li, Chengke Huang, Mengchun Chen, Yaoyao Dong, Lejing Lian, Wei Sun

**Affiliations:** ^1^The Second Affiliated Hospital and Yuying Children's Hospital of Wenzhou Medical University, Wenzhou 325027, Zhejiang, China; ^2^Wenzhou Medical University, Wenzhou 325035, Zhejiang, China; ^3^Ningbo Municipal Hospital of Traditional Chinese Medicine, Ningbo 315010, Zhejiang, China

## Abstract

*Corydalis decumbens*, a Traditional Chinese Medicine, has been widely used for the alternative and/or complementary therapy of hypertension, arrhythmias rheumatoid arthritis, sciatica, stroke, hemiplegia, paraplegia, and vascular embolism. The aim of this study was to determinate the potential effects of* Corydalis decumbens* on the five cytochrome P450 (CYP) enzyme activities (CYP1A2, CYP3A4, CYP2C9, CYP2C19, and CYP2D6) by cocktail approach. To evaluate whether concurrent use of* Corydalis decumbens* interferes with the effect of several prescription drugs, saline (control group) or* Corydalis decumbens* (XTW group) were administrated via gavage for 7 successive days. A probe cocktail solution (phenacetin, omeprazole, metoprolol, tolbutamide, and midazolam) was given 24 h after the last dose of saline or* Corydalis decumbens*. A specific and sensitive UHPLC–MS/MS method was validated for the determination of five substrates and their metabolites in control group and XTW group. Our results indicated that* Corydalis decumbens* could have inductive effects of CYP2C19 and inhibit the activities of CYP1A2 and CYP3A4. However,* Corydalis decumbens* had no significant influence on CYP2C9 and CYP2D6. The herb-drug interaction should require more attention by careful monitoring and appropriate drug dosing adjustments to the concurrent use of western medications which were metabolized by CYP1A2, CYP2C19, and CYP3A4 in human—*Corydalis decumbens*, Cytochrome P450, Cocktail, Pharmacokinetics, herb–drug interactions.

## 1. Introduction

Herbal medicine was gradually being used as an alternative and/or complementary treatment for serious diseases [1].* Corydalis decumbens* (Thunb.) Pers, a herbal medicine, was named “Xiatianwu” in China. Cultivation base of “Xiatianwu” was officially approved by Good Agriculture Practices (GAP), in China since 1980.* Corydalis decumbens*, also a Traditional Chinese Medicine (TCM), has been widely used for the treatment of hypertension, hemiplegia or cerebral embolism hemiplegia, paralytic stroke, rheumatic arthritis, and sciatica [2]. Earlier clinical and/or preclinical studies also have demonstrated that* Corydalis decumbens* (tablet/injection) has exerted the benefit effect on hypertension [3], rheumatoid arthritis [4], sciatica [5], trigeminal neuralgia [6], inhibiting platelet aggregation [7], and memory [8]. Thus, besides clinical doctor's recommendations, many patients take western medication in combination with* Corydalis decumbens* or other herbal medicine which they think is safe and often not inform self-medication to their primary physician [9–12]. However, there is a potential for herb–drug interactions between herbal medicine and western medicine [7]. The herb–drug interactions are implicated in pharmacokinetic and pharmacodynamic effects and may lead to serious adverse events or even death [13]. In order to avoid clinically insufficient benefits and/or unacceptable risks, it is important to discover and identify harmful combination interactions.

Cytochrome P450 (CYP), a superfamily of enzymes, is the main phase 1 enzyme system for the metabolism of various exogenous, endogenous components, and herbal substance [14, 15]. Among many CYP enzymes, CYP1A2, CYP2C9, CYP2C19, CYP2D6, and CYP3A4 are the major contributors to metabolizing a vast majority of widely known drugs [16, 17]. Inhibition or induction of specific enzymes has been considered as the mainly modulated factor for herb–drug interactions (HDIs), which can appear when herb and western medication are combined administration [18–20]. Therefore, it is essential to understand the inhibitive and inductive effects of CYP enzymes, in order to predict the potential HDI.

The present work was to evaluate the effects of* Corydalis decumbens* on the activities of CYP enzymes CYP1A2, CYP2C9, CYP2C19, CYP2D6, and CYP3A4. The cocktail approach was effectively used to monitor the activities of CYP enzymes and recognized as one of the specific analytical tools to study herb–drug interactions [21, 22]. Five probe drugs (CYP1A2 for phenacetin [23], CYP2C9 for tolbutamide [24], CYP2C19 for omeprazole [25], CYP2D6 for metoprolol [26], and CYP3A4 for midazolam [27]) were analysed in rat plasma with a specific and sensitive UHPLC–MS/MS method. Several studies have demonstrated that rat CYPs (1A2, 2C6, 2D1, 2D2, and 3A1/2) are homologous to human CYPs (1A2, 2C9, 2C19, 2D6, and 3A4), respectively [17, 19, 28]. Therefore, the results from rats could be extrapolated to human in clinical use [17, 19]. Five CYP enzymes activities were analysed by comparing pharmacokinetics of corresponding probe drugs between XTW and control treatment groups. We hope that our results will be helpful for avoiding the insufficient benefits and serious adverse effects of herb–drug interactions between* Corydalis decumbens* and western medicine.

## 2. Materials and Methods

### 2.1. Chemicals

Phenacetin, omeprazole, metoprolol, tolbutamide, midazolam (purity > 98%), and carbamazepine (internal standard, IS) were provided by Sigma–Aldrich Co. (St. Louis, MO, USA).* Corydalis decumbens* injection was obtained from Jiang Xi Herbi-sky Co., Ltd. (JiangXi, China). A Milli–Q system (Millipore Co., USA) was used to produce ultrapure water. Other chemical reagents (analytical grade) used were from standard chemical suppliers.

### 2.2. Animals

Male Sprague–Dawley rats (n=12, weighing 220±20 g) were obtained from Laboratory Animal Research Center of Wenzhou Medical University (Wenzhou, China). The rats were kept in house cages with a temperature 22±2°C, humidity 50±5%, and light–dark (12 h/12 h) cycle. They were maintained in house cages for at least one week prior to study pharmacokinetics experimentation and fed with food and drinking water freely. All experimental procedures in present study were approved strictly by the Animal Care and Use Committee of Wenzhou Medical University.

### 2.3. Apparatus and UHPLC–MS/MS Conditions

To analyse the mixed compounds, the UHPLC–MS/MS was employed. Chromatographic analysis was executed by an Agilent 1290 UHPLC system. The mass spectrometry was Agilent 6420 Series Triple Quadrupole Tandem Mass Spectrometer (Santa Clara, CA, USA) equipped with an electrospray ionization source in the positive-ion mode. MassHunter Agilent Software (version B.07.00) was used for setting instrument condition parameters and analysing data.

Separation of five probes and the IS was based on the conditions described previously [17]. They were separated using a 2.1 × 50 mm, 1.8 *μ*m particle, Agilent ZORBAX Eclipse Plus C18 Rapid Resolution HD column at a constant temperature of 30°C. Formic acid–water, 1/1000, v/v (mobile phase A), and acetonitrile (mobile phase B) were prepared as initial mobile phases, which were ultrasonically degassed before use. A gradient elution program was as follows: 0–0.3 min (30% B); 0.3–1.3 min (30%–50% B); 1.3–1.8 min (50%–95%B); 1.8–2.8 min (95% B). The posttime was 1.0 min for equilibration of the column, and the total run time was 3.8 min. The flow rate was 0.4 mL·min^−1^.

Nitrogen was considered as desolvation gas (10 L/h). The nebuliser pressure and desolvation temperature of drying gas (both nitrogen) flow were adjusted to 45 psi and 350°C, respectively. The capillary voltage was set to 4 KV. The quantitative analysis of target ions was performed in multiple reaction monitoring (MRM) mode with m/z 180.1→109.9 for phenacetin, m/z 346.1→135.9 for omeprazole, m/z 268.2→115.9 for metoprolol, m/z 271.1→91.0 for tolbutamide, m/z 326.1→ 290.8 for midazolam, and m/z 237.1→194.0 for the IS.

### 2.4. Pharmacokinetics

The study was conducted in accordance with the BCPT policy for experimental and clinical studies [29]. The above 12 male Sprague–Dawley rats were randomly divided into 2 groups:* Corydalis decumbens* treatment group (XTW group) (n = 6) and control group (n = 6).* Corydalis decumbens* and saline (5 mL/kg via gavage, i.g.) were administrated for 7 successive days. A probe cocktail solution was prepared by phenacetin (10 mg/kg), omeprazole (10 mg/kg), metoprolol (10 mg/kg), tolbutamide (1 mg/kg), and midazolam (10 mg/kg) diluted with saline. The probe cocktail solution (4 mL/kg via i.g.) was given 24 h after the last dose of saline or* Corydalis decumbens*.

Tail vein blood samples (0.25-0.3 mL) were collected into 1.5 mL heparinized polyethylene tubes at 0.17, 0.5, 1, 2, 3, 4, 6, 8, 12, 24, 48, and 72 h, after probe drugs administration. The blood samples were centrifuged at 10,000 g for 10 min and plasma layers were dispatched aliquots of 100 *μ*L and frozen at –80°C until analysis.

### 2.5. Statistical Analysis

Drug and statistics (DAS) software (version 3.0) was used to calculate the pharmacokinetic parameters. The results were expressed as a mean ± standard deviation (SD). All analyses for the main pharmacokinetic parameters of the 2 groups were performed with the IBM SPSS software system statistics (version 23.0) by use of Student's t-test. Statistical significance was accepted if a value of p < 0.05.

## 3. Results

### 3.1. Method Validation

The regression type of each analyte in a certain range, correlation coefficient, and calibrations were shown in Table 1. The calibration curves showed good linearity over the selected concentration in all analyte samples with a correlation coefficient (R^2^) > 0.990. The lower limit of quantification(LLOQ) was 5.43 ng/mL for phenacetin, 3.43 ng/mL for tolbutamide, 5.65 ng/mL for omeprazole, 5.33 ng/mL for metoprolol, and 4.46 ng/mL for midazolam.

**Figure 1:**
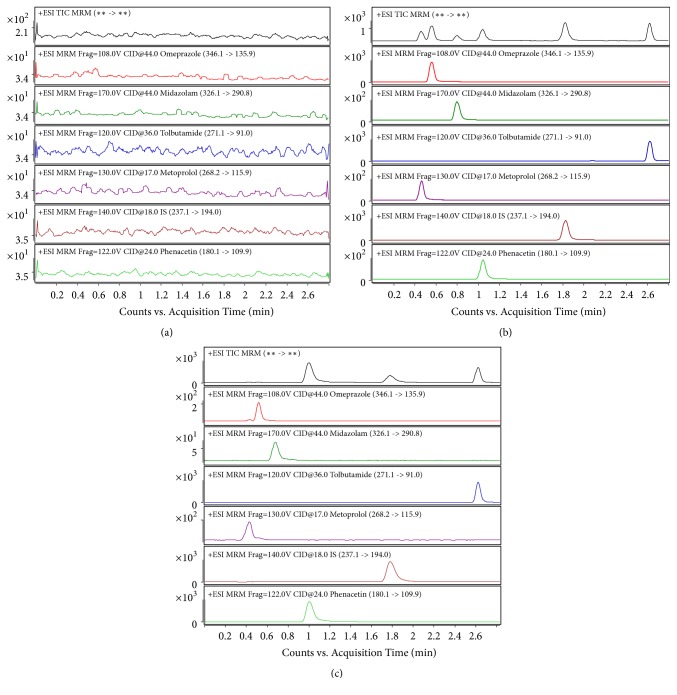
MRM chromatograms of (a) blank plasma samples, (b) blank plasma samples spiked with the probe drugs, and (c) plasma sample obtained from rat after administration of five cocktail probe drugs.

As shown in Figure 1, the retention times of relevant analytes (phenacetin, tolbutamide, omeprazole, metoprolol, and midazolam) and IS determined by a UHPLC-MS/MS method in rat plasma were 1.035, 2.649, 0.529, 0.448, 0.715, 1.827 min, respectively. Meanwhile, there were no interferences of endogenous interfering peaks near the relative retention time for analytes in EIC chromatograms. The dates of precision, accuracy, recovery, and matrix effect of five probes were summarized in Table 2. The intraday and interday precisions ranged from 2.25% to 10.85% and 2.90% to 9.74%, respectively. The intraday and interday accuracies changed from 95.56% to 106.24% and 97.26% to 103.97%, respectively. The extraction recoveries for phenacetin, tolbutamide, omeprazole, metoprolol, and midazolam were larger than 83.5%. The matrix effects for all analytes ranged from 95.26% to 102.59%. Abovementioned results were collected in Table 2.

### 3.2. Effect of* Corydalis decumbens* on the CYP1A2 Activity

**Figure 2:**
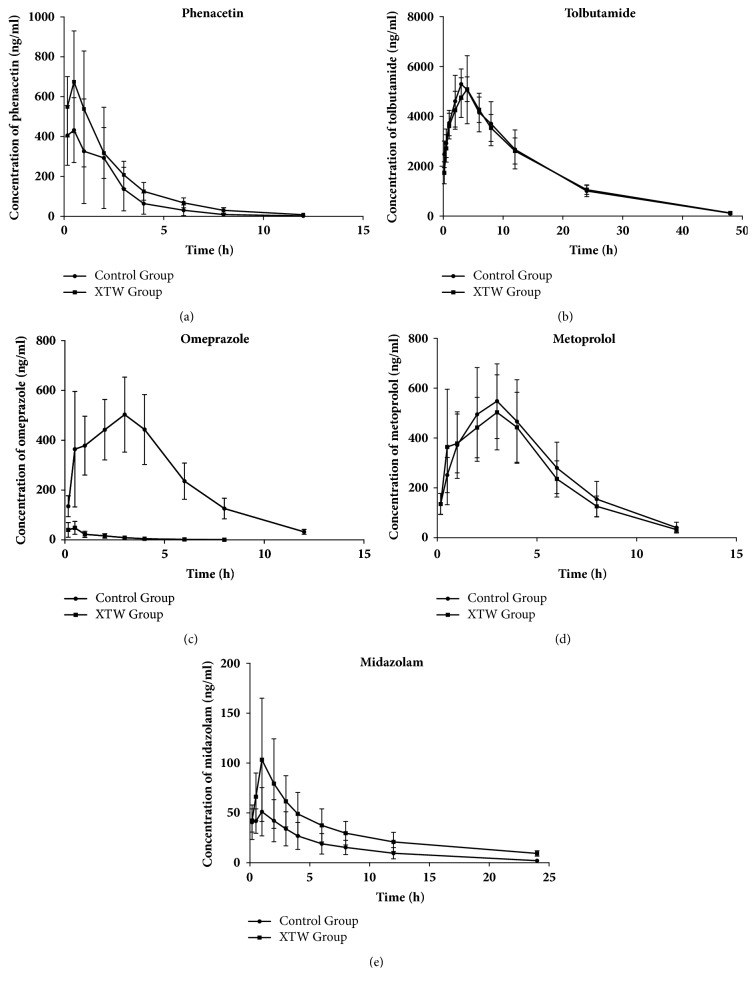
Plasma concentration-time curves of probe drug (phenacetin, tolbutamide, omeprazole, metoprolol, and midazolam). Control Group: the metabolism of the probe drugs in vivo. XTW Group: the metabolism of the probe drugs in vivo after rats received intraperitoneal injection of* Corydalis decumbens*.

Pharmacokinetic profiles of phenacetin in two different treatment groups were used to depict CYP1A2 activity. As shown in Figure 2(a) and Tables 3(a) and 3(b), XTW group significantly prolonged T_1/2_ and increased MRT_(0–t)_ and MRT_(0–*∞*)_ (P<0.05, P<0.01), when compared with control group. Other pharmacokinetic parameters (C_max_, AUC and CLz/F) of phenacetin in the XTW group showed no obvious differences, comparing with control group (P>0.05). These indicated that* Corydalis decumbens* might inhibit the activity of CYP1A2.

### 3.3. Effect of* Corydalis decumbens* on the CYP2C9 Activity

Tolbutamide's pharmacokinetic parameters in two different groups were expressed in Figure 2(b) and Tables 3(a) and 3(b). There were no obvious differences in the pharmacokinetic parameters in XTW and control groups, which indicated that* Corydalis decumbens* did not influence the activity of CYP2C9 in blood samples.

### 3.4. Effect of* Corydalis decumbens* on the CYP2C19 Activity

The activity of CYP2C19 was evaluated by measuring the omeprazole's pharmacokinetic parameters in different groups (Figure 2(c), Tables 3(a) and 3(b)). T_1/2_, T_max_, MRT_(0–t)_, MRT_(0–*∞*)_, AUC_(0–t)_, and AUC_(0–*∞*)_ values of omeprazole in rats treated by* Corydalis decumbens* decreased significantly (P<0.05, P<0.01). The value of CLz/F for omeprazole was increased significantly with* Corydalis decumbens* treatment, comparing with control group (P<0.05). These date demonstrated that metabolism of omeprazole was obviously accelerated by* Corydalis decumbens* treatment, and it had potential to induct the CYP2C19 activity in blood samples.

### 3.5. Effect of* Corydalis decumbens* on the CYP2D6 Activity

The activity of CYP2D6 was assessed by comparing the metoprolol's pharmacokinetic parameters between XTW and control groups. As shown in Figure 2(d) and Tables 3(a) and 3(b), the effect of* Corydalis decumbens* on the all pharmacokinetic parameters of metoprolol was no significant change, comparing with control group. Thus, metoprolol's pharmacokinetic behaviors demonstrated that the CYP2D6 activity might not be inhibited or inducted by* Corydalis decumbens*.

### 3.6. Effect of* Corydalis decumbens* on the Activity of CYP3A4

As shown in Figure 2(e) and Tables 3(a) and 3(b), pharmacokinetic behaviors of midazolam were significant differences in XTW and control treatment groups. The value of T_1/2_ and C_max_ for midazolam was increased significantly with* Corydalis decumbens* treatment (P<0.05, P<0.01), and the value of CLz/F was decreased obviously (P<0.05). It indicated that* Corydalis decumbens* might prolong the elimination and increase midazolam absorbed into blood. Meanwhile, other pharmacokinetic parameters (MRT_(0–t)_, MRT_(0–*∞*)_, AUC_(0–t)_, and AUC_(0–*∞*)_ were increased significantly (P<0.01) with* Corydalis decumbens* treatment. These date showed that the activity of CYP3A4 might be inhibited by* Corydalis decumbens*.

## 4. Discussion

In the present study, the potential effects of* Corydalis decumbens* on the activities of CYP enzymes (CYP1A2, CYP2C9, CYP2C19, CYP2D6, and CYP3A4) were detected by a novel UHPLC-MS/MS method. The method was validated for the determination of novel “cocktail” in linearity, accuracy, precision, selectivity, recovery, matrix effect, and stability and successfully applied in the pharmacokinetic study. Our results indicated that the activities of CYP1A2 and CYP3A4 might be inhibited by* Corydalis decumbens*. And, the activity of CYP2C19 was induced by* Corydalis decumbens*. However, there were no significant differences in pharmacokinetics dates of tolbutamide and metoprolol between two different groups, which demonstrated that the activities of CYP2C9 and CYP2D6 were not obviously influenced by* Corydalis decumbens*.

Recently, herbal medicine is an increasingly common form of alternative and/or complementary therapy in several countries (e.g., China, United States, Japan, Korea, Sweden, France, Germany, and Australia) [1]. Often herbal products are regulated as dietary supplements and patients typically think it is safer than pharmaceutical drugs [30–32]. But, several studies specifically defined that the concurrent use of herbal medicines and prescription drugs may trigger the potentiality of herb–drug interactions [33, 34]. These interactions may cause the inhibition or induction of specific CYP enzymes and elicit pharmacokinetic and pharmacodynamic mechanisms which may result serious clinical consequences [18]. Thus, it is important to update and improve pharmacist's and physician's knowledge of HDI to properly counsel and avoid improper concurrent use of herbal medicine and prescription drugs. Chinese Pharmacopoeia recorded that* Corydalis decumbens* plays an important role in terms of hypertension, arrhythmias rheumatoid arthritis, sciatica, stroke, hemiplegia, paraplegia, and vascular embolism [2]. However, there are limited literature and data about HDI in* Corydalis decumbens*. It is important to evaluate whether concurrent administration of herbs may interfere with the effect of drugs.

In the treatment of hypertension,* Corydalis decumbens* could reduce blood pressure by relaxing vascular smooth muscle and reducing peripheral resistance [3]. Propranolol, an antihypertensive medication of the beta blocker class, is mainly metabolized by CYP1A2 and CYP2D6 [35]. Nifedipine, a calcium channel blocker, is mainly metabolized by CYP3A4 [36]. In the present study, the activities of CYP1A2 and CYP3A4 might be inhibited by* Corydalis decumbens*. Thus, the concomitant use of* Corydalis decumbens* can interfere with the pharmacokinetics or pharmacodynamics of propranolol/nifedipine. As a result, the dosage of these drugs may be decreased or the dosing interval be increased.

Rheumatoid arthritis is a systemic immune disease characterized by noninfectious inflammation of the joints and tissues surrounding the joints [37]. Several clinical studies indicated that* Corydalis decumbens* tablets have a benefit effect on alleviating the clinical symptoms of rheumatoid arthritis, with little side effects [3, 38]. Diclofenac, a nonsteroidal anti-inflammatory drug, is commonly metabolized by CYP2C9 [7]. The present results showed that the activity of CYP2C9 was not obviously influenced by* Corydalis decumbens*. There was no interaction with the concurrent administration of* Corydalis decumbens* and diclofenac. When a patient requires a concomitant drug to treat rheumatoid arthritis, the therapy may produce synergistic effects and should be favored.

Furthermore,* Corydalis decumbens* were usually used as a complementary medicine for sciatica due to its pharmacological effects of Tongluo analgesics [2]. Naproxen is a nonsteroidal anti-inflammatory drug (NSAID), which relieves pain, fever, swelling, and stiffness. Naproxen is also metabolized by CYP2C9 [39, 40]. And, the concurrent use of* Corydalis decumbens* and naproxen is recommended.

Last but not least, cerebrovascular disease is defined as a general term for a group of diseases, which includes stroke, hemiplegia, paraplegia, and vascular embolism, caused by various acute and chronic cerebrovascular diseases. Anticoagulant drugs and antiplatelet agent play an indispensable role in treatment of these diseases, because platelet activation, adhesion, and aggregation are one of the initiating factors of intravascular thrombosis. Warfarin, a vitamin K-antagonist drug, is mainly metabolized by cytochrome P450 enzymes CYP2C9 and CYP1A2 [41–43]. Our results indicated that the activity of CYP2C9 was not obviously influenced by* Corydalis decumbens*, but CYP1A2 was inhibited. The herb–drug interaction could prolong the elimination and increase warfarin absorbed into blood, which may increase bleeding events. Meanwhile, warfarin has a narrow therapeutic index, especially when combined with potentially interacting drugs. Thus, it is a necessary reminder that the dosage of warfarin can reduce and the dosing interval can increase, when the patients receive long-term concurrent use of* Corydalis decumbens* and warfarin. Clopidogrel is an effective antiplatelet agent useful for the treatment of ischemic cerebrovascular disease by blocking ADP receptors on the platelet membrane [44]. Clopidogrel is metabolized by cytochrome P450 enzymes CYP2C19 [45]. The present study also found that the activities of CYP2C19 were inducted by* Corydalis decumbens*. The clearance rate for clopidogrel may increase significantly with* Corydalis decumbens* treatment. The herb-drug interaction may produce quick therapeutic effect and faster loss of efficacy. When a patient needs a coprescription, it is properly counseled that the dosage of clopidogrel may be increased or the dosing interval may be decreased.

## 5. Conclusions

The cocktail approach was effectively used as a potential screening tool for effects in vivo HDI. Five probe drugs phenacetin, tolbutamide, omeprazole, metoprolol, and midazolam were selected as specific substrates for rat CYP1A2, CYP2C6, CYP2D1, CYP2D2, and CYP3A1/2, respectively. Also, these probe drugs more often relate to human CYP1A2, CYP2C9, CYP2C19, CYP2D6, and CYP3A4, even though they are given to rats. Thus, the effects of* Corydalis decumbens* determined in rat are also useful for predicting clinical HDIs. Our results indicated that* Corydalis decumbens* could induce the CYP2C19 activity and inhibit the activities of CYP1A2 and CYP3A4. During the concurrent use of* Corydalis decumbens* with western medications which were extensively metabolized by CYP1A2, CYP3A4, and CYP2C19 in human, the herb–drug interaction should require more attention by careful monitoring and appropriate drug dosing adjustments to avoid some unacceptable risks and reduce drug accumulation or ineffective treatment.

## Figures and Tables

**Table 1:** The calibration curve of phenacetin, metoprolol, midazolam, omeprazole, and tolbutamide in plasma (n=6).

Analytes	Calibration curves	R^2^	linear range (ng·mL)
Phenacetin	Y=0.502240*∗*X+0.010662	R^2^=0.9984	2.5-1000ng/ml
Metoprolol	Y=0.301110*∗*X+0.063879	R^2^=0.9903	2.5-1000ng/ml
Midazolam	Y=0.219790*∗*X+0.011036	R^2^=0.9965	0.5-200ng/ml
Omeprazole	Y=0.556111*∗*X+0.030624	R^2^=0.9970	1-1000ng/ml
Tolbutamide	Y=0.536867*∗*X+0.081507	R^2^=0.9952	20-8000ng/ml

**Table 2:** Precision, accuracy, extraction recovery, and matrix effect of five probes in rat plasma (n=6).

Analytes	Concentration added (ng/mL)	Intra-day		Inter-day		Recovery (%)	Matrix effect (%)
		RSD (%)	RE (%)	RSD (%)	RE (%)		
Phenacetin	5	4.93	2.10	4.90	-0.19	83.50	98.41
	100	7.02	1.69	8.02	2.78	84.71	97.97
	500	2.42	-1.39	5.06	-0.93	87.35	102.59
Metoprolol	5	10.85	-0.88	9.74	-0.39	88.98	95.26
	100	8.27	-3.41	9.16	-2.74	90.76	97.67
	500	4.70	1.86	5.90	0.42	87.24	97.27
Midazolam	2.5	8.01	4.40	8.82	0.68	85.62	95.46
	25	8.60	-0.48	9.22	-2.21	84.02	96.47
	100	5.65	0.77	7.56	-0.28	87.48	97.82
Omeprazole	5	5.69	-0.58	6.91	1.72	85.93	98.78
	50	6.45	4.28	5.15	-2.18	88.93	95.91
	500	2.25	6.24	2.90	1.66	86.95	100.34
Tolbutamide	100	2.41	-4.44	8.12	3.97	85.82	98.94
	500	4.17	1.87	4.66	-0.73	87.43	95.97
	5000	3.28	0.41	4.23	-1.19	86.85	99.18

**Table:** (a) Main pharmacokinetic parameters of six in rats (n=6, mean±SD)

Probe drugs	Parameters	T_1/2_z, h	CLz/F, L/h/kg	C_max_, ng/ml	T_max_,h
Phenacetin	Control	1.364±0.189	12.167±7.187	468.492±174.355	0.473±0.304
	XTW	2.005±0.084*∗∗*	6.489±3.258	715.237±216.924	0.528±0.266
Metoprolol	Control	2.175±0.350	3.256±1.240	595.115±176.562	3.000±0.894
	XTW	2.137±0.109	3.353±0.672	602.857±164.967	2.583±1.201
Midazolam	Control	5.426±0.323	31.808±13.079	57.127±22.704	0.723±0.429
	XTW	6.941±0.770*∗∗*	13.913±4.180*∗*	126.572±55.454*∗*	1.167±0.683
Omeprazole	Control	2.137±0.109	3.353±0.672	602.857±164.967	2.583±1.200
	XTW	1.435±0.222*∗∗*	160.865±125.736*∗*	53.273±28.328*∗∗*	0.335±0.181*∗∗*
Tolbutamide	Control	7.928±0.501	0.123±0.024	5753.637±1135.602	3.167±0.753
	XTW	8.196±0.400	0.122±0.016	5327.587±586.093	3.667±0.516

*∗*: significantly different from control, p<0.05. *∗∗*: significantly different from control, p<0.01.

**Table:** (b) Main pharmacokinetic parameters of six in rats (n=6, mean±SD)

Probe drugs	Parameters	MRT_(0–t)_,h	MRT_(0–*∞*)_,h	AUC_(0–t)_, ng/ml·h	AUC_(0–*∞*)_, ng/ml·h
Phenacetin	Control	1.881±0.364	1.934±0.336	1158.538±790.101	1162.355±789.191
	XTW	2.460±0.294*∗*	2.630±0.336*∗∗*	1778.848±652.752	1802.074±657.140
Metoprolol	Control	4.191±0.387	4.629±0.449	3266.639±1033.756	3399.903±1078.993
	XTW	4.003±0.460	4.372±0.524	2995.480±681.621	3098.636±703.838
Midazolam	Control	6.114±0.409	7.178±0.583	354.710±170.609	371.116±182.508
	XTW	7.236±0.701*∗∗*	9.330±1.093*∗∗*	717.013±231.178*∗*	780.230±260.146*∗*
Omeprazole	Control	4.004±0.460	4.372±0.524	2995.480±681.621	3098.636±703.838
	XTW	1.747±0.160*∗∗*	1.941±0.176*∗∗*	88.440±45.975*∗∗*	90.921±47.286*∗∗*
Tolbutamide	Control	11.563±0.526	12.339±0.505	82485.815±16562.483	83822.392±16723.482
	XTW	11.897±0.709	12.742±0.850	81505.311±10227.159	82972.008±10446.975

*∗*: significantly different from control, p<0.05. *∗∗*: significantly different from control, p<0.01.

## Data Availability

The data used to support the findings of this study are available from the corresponding author upon request

## References

[1] de Smet P. A. G. M. (2002). Herbal remedies. *The New England Journal of Medicine*.

[2] Wu C., Yan R., Zhang R. (2013). Comparative pharmacokinetics and bioavailability of four alkaloids in different formulations from Corydalis decumbens. *Journal of Ethnopharmacology*.

[3] Wang Q., Li Z., Yang Z. (2016). New alkaloids with anti-inflammatory activities from Corydalis decumbens. *Phytochemistry Letters*.

[4] Yu T., Li Z., Wang J. (2007). Clinical observation on 43 cases of rheumatoid arthritis treated with compound Corydalis Decumbens tablets. *Chinese Journal of Traditional Medical Science and Technology*.

[5] Song Q. (2008). Therapeutic effect of Corydalis Decumbens acupoint injection on sciatica. *Chinese Practical Journal of Rural Doctor*.

[6] Chen L. (2007). Clinical observation on 42 cases of trigeminal neuralgia treated by acupoint injection of *Corydalis Decumbens* injection and chinese herbal medicine. *Chinese Practical Journal of Rural Doctor*.

[7] Shimamoto J., Ieiri I., Urae A. (2000). Lack of differences in diclofenac (a substrate for CYP2C9) pharmacokinetics in healthy volunteers with respect to the single CYP2C9∗3 allele. *European Journal of Clinical Pharmacology*.

[8] Yu L., Yu S., Gong Q. (2006). *Corydalis Decumbens* Pers Injection on Ang-1 expression of hippocampus in vascular dementia rats. *Chin Tradit Patent Med*.

[9] Astin J. A., Marie A., Pelletier K. R., Hansen E., Haskell W. L. (1998). A review of the incorporation of complementary and alternative medicine by mainstream physicians. *JAMA Internal Medicine*.

[10] Eisenberg D. M., Davis R. B., Ettner S. L. (1998). Trends in alternative medicine use in the United States, 1990–1997: results of a follow-up national survey. *The Journal of the American Medical Association*.

[11] Kaufman D. W., Kelly J. P., Rosenberg L., Anderson T. E., Mitchell A. A. (2002). Recent patterns of medication use in the ambulatory adult population of the United States: the Slone survey. *Journal of the American Medical Association*.

[12] Kelly J. P., Kaufman D. W., Kelley K., Rosenberg L., Anderson T. E., Mitchell A. A. (2005). Recent trends in use of herbal and other natural products. *JAMA Internal Medicine*.

[13] Wienkers L. C., Heath T. G. (2005). Predicting in vivo drug interactions from in vitro drug discovery data. *Nature Reviews Drug Discovery*.

[14] Michielan L., Terfloth L., Gasteiger J., Moro S. (2009). Comparison of multilabel and single-label classification applied to the prediction of the isoform specificity of cytochrome p450 substrates. *Journal of Chemical Information and Modeling*.

[15] Rendic S. (2002). Summary of information on human CYP enzymes: Human P450 metabolism data. *Drug Metabolism Reviews*.

[16] Rendic S., Guengerich F. P. (2015). Survey of human oxidoreductases and cytochrome P450 enzymes involved in the metabolism of xenobiotic and natural chemicals. *Chemical Research in Toxicology*.

[17] Sun W., Wang Z., Chen R. (2017). Influences of Anlotinib on Cytochrome P450 Enzymes in Rats Using a Cocktail Method. *BioMed Research International*.

[18] Al-Ramahi R., Jaradat N., Shalalfeh R. (2015). Evaluation of potential drug- herb interactions among a group of Palestinian patients with chronic diseases. *BMC Complementary and Alternative Medicine*.

[19] Geng T., Si H., Kang D. (2015). Influences of Re du Ning Injection, a traditional Chinese medicine injection, on the CYP450 activities in rats using a cocktail method. *Journal of Ethnopharmacology*.

[20] Han Y.-L., Li D., Ren B. (2012). Evaluation of impact of Herba Erigerontis injection, a Chinese herbal prescription, on rat hepatic cytochrome P450 enzymes by cocktail probe drugs. *Journal of Ethnopharmacology*.

[21] Wang X., Han A., Wen C. (2013). The effects of H2s on the activities of CYP2B6, CYP2D6, CYP3A4, CYP2C19 and CYP2C9 in vivo in rat. *International Journal of Molecular Sciences*.

[22] Wang Z., Sun W., Lin Z. (2019). A UHPLC-MS/MS method coupled with liquid-liquid extraction for the quantitation of phenacetin, omeprazole, metoprolol, midazolam and their metabolites in rat plasma and its application to the study of four CYP450 activities. *Journal of Pharmaceutical and Biomedical Analysis*.

[23] Bartoli A., Xiaodong S., Gatti G., Cipolla G., Marchiselli R., Perucca E. (1996). The influence of ethnic factors and gender on CYP1A2-mediated drug disposition: A comparative study in Caucasian and Chinese subjects using phenacetin as a marker substrate. *Therapeutic Drug Monitoring*.

[24] Hemeryck A., De Vriendt C., Belpaire F. M. (1999). Inhibition of CYP2C9 by selective serotonin reuptake inhibitors: In vitro studies with tolbutamide and (S)-warfarin using human liver microsomes. *European Journal of Clinical Pharmacology*.

[25] Kanazawa H., Okada A., Higaki M., Yokota H., Mashige F., Nakahara K. (2003). Stereospecific analysis of omeprazole in human plasma as a probe for CYP2C19 phenotype. *Journal of Pharmaceutical and Biomedical Analysis*.

[26] Werner U., Werner D., Rau T., Fromm M. F., Hinz B., Brune K. (2003). Celecoxib inhibits metabolism of cytochrome P450 2D6 substrate metoprolol in humans. *Clinical Pharmacology & Therapeutics*.

[27] Gupta P., Alvey C., Wang R. (2012). Lack of effect of tofacitinib (CP-690,550) on the pharmacokinetics of the CYP3A4 substrate midazolam in healthy volunteers: Confirmation of in vitro data. *British Journal of Clinical Pharmacology*.

[28] Videau O., Pitarque S., Troncale S. (2012). Can a cocktail designed for phenotyping pharmacokinetics and metabolism enzymes in human be used efficiently in rat?. *Xenobiotica*.

[29] Tveden-Nyborg P., Bergmann T. K., Lykkesfeldt J. (2018). Basic & Clinical Pharmacology & Toxicology Policy for Experimental and Clinical studies. *Basic Clin Pharmacol Toxicol*.

[30] Goldstein L. H., Elias M., Ron-Avraham G. (2007). Consumption of herbal remedies and dietary supplements amongst patients hospitalized in medical wards. *British Journal of Clinical Pharmacology*.

[31] Klepser T. B., Doucette W. R., Horton M. R. (2000). Assessment of patients' perceptions and beliefs regarding herbal therapies. *Pharmacotherapy*.

[32] Mattsson K., Nilsson I. (2002). Herbal preparations have both effects and side effects. Widespread usage dictates knowledge among physicians. *Läkartidningen*.

[33] Izzo A. A., Ernst E. (2001). Interactions between herbal medicines and prescribed drugs: A systematic review. *Drugs*.

[34] Izzo A. A., Ernst E. (2009). Interactions between herbal medicines and prescribed drugs. *Drugs*.

[35] Masubuchi Y., Hosokawa S., Horie T. (1994). Cytochrome P450 isozymes involved in propranolol metabolism in human liver microsomes: The role of CYP2D6 as ring-hydroxylase and CYP1A2 as N- desisopropylase. *Drug Metabolism and Disposition*.

[36] Fuhr U., Beckmann-Knopp S., Jetter A., Lück H., Mengs U. (2007). The effect of silymarin on oral nifedipine pharmacokinetics. *Planta Medica*.

[37] Katz S. I. (2015). Commentary: Setting priorities for research funding at the National Institute of Arthritis and Musculoskeletal and Skin Diseases (NIAMS). *Journal of the American Academy of Dermatology*.

[38] Zhao H., Huang Y., Zhang J. (2011). Clinical observation of no compound knee osteoarthritis in Corydalis Decumbens. *Strait Pharmaceutical Journal*.

[39] Song Q. (1995). Observation of sciatica treated with point injection of XIA TIAN WU contrast analysis of 78 case. *Guangxi Journal of Traditional Chinese Medicine*.

[40] McGinnity D. F., Tucker J., Trigg S., Riley R. J. (2005). Prediction of CYP2C9-mediated drug-drug interactions: A comparison using data from recombinant enzymes and human hepatocytes. *Drug Metabolism and Disposition*.

[41] Van Dijk K. N., Plat A. W., Van Dijk A. A. C. (2004). Potential interaction between acenocoumarol and diclofenac, naproxen and ibuprofen and role of CYP2C9 genotype. *Thrombosis and Haemostasis*.

[42] Kaminsky L. S., Zhang Z.-Y. (1997). Human P450 metabolism of warfarin. *Pharmacology & Therapeutics*.

[43] Takahashi H., Echizen H. (2003). Pharmacogenetics of CYP2C9 and interindividual variability in anticoagulant response to warfarin. *The Pharmacogenomics Journal*.

[44] Wadelius M., Sörlin K., Wallerman O. (2004). Warfarin sensitivity related to CYP2C9, CYP3A5, ABCB1 (MDR1) and other factors. *The Pharmacogenomics Journal*.

[45] Holmes M. V., Perel P., Shah T., Hingorani A. D., Casas J. P. (2011). CYP2C19 genotype, clopidogrel metabolism, platelet function, and cardiovascular events: a systematic review and meta-analysis. *Journal of the American Medical Association*.

